# Phakic Intraocular Lenses as a Potential Treatment for Pigment Dispersion Syndrome

**DOI:** 10.7759/cureus.108051

**Published:** 2026-04-30

**Authors:** Matthew Hirabayashi, Ayorinde Cooley, Fatma Shakarchi, Anthony Vanrachack, Laurence Ducker, Christopher Shelby, Wyche T Coleman, Gregory D Parkhurst, Stephen LoBue

**Affiliations:** 1 Ophthalmology, Parkhurst NuVision LASIK Eye Surgery, San Antonio, USA; 2 Ophthalmology, Willis-Knighton Medical Center, Shreveport, USA; 3 Ophthalmology, Mason Eye Clinic, Columbia, USA

**Keywords:** evo icl, glaucoma, phakic iol, pigment dispersion glaucoma, pigment dispersion syndrome

## Abstract

Pigment dispersion syndrome/pigment dispersion glaucoma (PDG) has no definitive cure and can cause vision loss in young myopes. Backward bowing of the iris can lead to rubbing of the pigmented epithelium against lens zonules and accumulation of pigment, obstructing aqueous humor outflow. We describe a case of a 37-year-old myopic man with PDG who presented to our clinic for a refractive surgery evaluation. The manifest refraction was -6.50 -0.50 x 107 in the right eye (oculus dexter (OD)) and -6.00 in the left eye (oculus sinister (OS)), which was correctable to 20/20 in both eyes (oculus uterque (OU)). Intraocular pressure (IOP) was 18 and 19 on latanoprost. Slit lamp examination revealed radial transillumination defects OU with superior and inferior thinning on nerve optical coherence tomography. Corneal topography and tomography were normal with a minimum pachymetry of 529 OD and 534 OS. Phakic intraocular lenses (IOLs; e.g., EVO implantable collamer lens (ICL)) were selected as a treatment option as they would fully correct the refractive error while providing a physical barrier between the zonules and posterior iris. On post-op day 1, the patient’s uncorrected distance vision was 20/20+2 OD and 20/15 OS, with IOPs of 11 and 9 by Goldmann applanation tonometry, respectively. The ICL vault had an OD of 744 µm and an OS of 766 µm. At post-op month 1, reconfiguration of the iris contour from concave to parallel was observed. A six-month follow-up of latanoprost demonstrated stable IOP of 11 OD and 12 OS with no signs of worsening PDG in slit lamp examination or glaucoma imaging. Although larger and longer studies are needed, the newer generation of phakic IOLs, such as the EVO ICL, may have a therapeutic role in pigment dispersion by changing the iris configuration and creating a mechanical barrier between the zonules and the posterior iris.

## Introduction

Pigment dispersion syndrome (PDS) is characterized by the excessive spread of iris pigment in anterior segment structures, including the corneal endothelium and trabecular meshwork. Pigment is released through chaffing of the posterior iris against the anterior zonules of the lens. This contact is proposed to be driven by the presence of a concave iris contour [1].

The prevalence of PDS is uncommon, with approximately 25,000-220,000 persons affected in the United States [2]. Accumulation of pigment within the trabecular meshwork can impede aqueous humor outflow, leading to an increase in intraocular pressure (IOP). Progression of this condition can cause pigmentary glaucoma, a form of secondary open-angle glaucoma.

Treatment of pigmentary glaucoma involves lowering IOP with glaucoma medications, laser trabeculoplasty, or laser peripheral iridotomy (LPI). Laser iridotomy has been employed to change iris configuration to decrease PDS with varied success. Numerous cases have documented worsening PDS even with a patent LPI [3]. Additionally, LPI is not without unwanted side effects, including elevated IOP, epithelial defects, and visual dysfunction, including glare or blurred vision [4,5].

The implantable collamer lens (ICL) is a phakic intraocular lens (IOL) designed to correct a range of refractive errors. ICLs are implanted within the posterior chamber between the crystalline lens and posterior iris, and can subsequently change iris configuration by providing physical support to the posterior surface of the iris, flattening its curvature [6,7]. The recent EVO ICL (STAAR Surgical, Monrovia, CA) has been shown to maintain long-term changes in iris configuration without significant postoperative complications [7,8]. Thus, we propose that the EVO ICL could be used as a therapeutic tool in pigment dispersion glaucoma (PDG) by changing the iris configuration by creating a mechanical barrier between the lens zonules and posterior iris.

## Case presentation

A 37-year-old myopic man presented to our clinic seeking independence from glasses and contact lenses. His preoperative manifest refraction was -6.50 -0.50 x 107 in the right eye (oculus dexter (OD)) and -6.00 in the left eye (oculus sinister (OS)), which was correctable to 20/20 in both eyes (oculus uterque (OU)). His cycloplegic refraction was unchanged from the manifest refraction. A contact lens overrefraction was -1.50 -0.50 x 105 with -4.25 contact lens OD and -1.25 with a -4.25 contact lens OS, correctable to 20/20+2 and 20/15-2, respectively. His uncorrected vision was counting fingers 2 ft OD and 20/400 OS.

**Figure 1:**
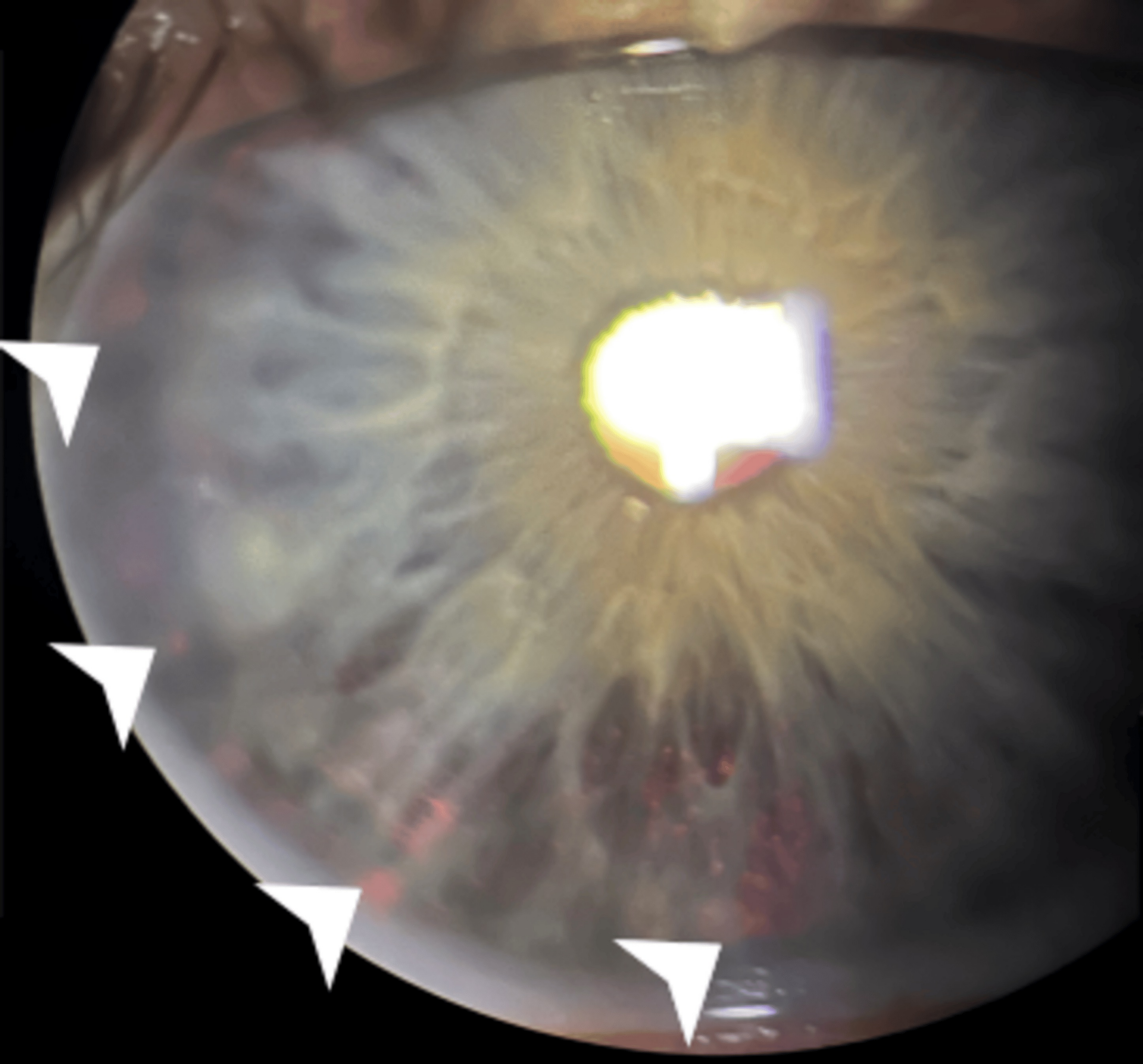
Preoperative slit-lamp photograph displaying the patient’s transillumination defects (white arrows)

The patient stated a previous diagnosis of PDG, characterized by elevated IOP, radial iris transillumination defects, and glaucomatous optic nerve changes (retinal nerve fiber layer (RNFL) thinning) in the setting of myopia, for which latanoprost had been started several years prior. IOP on latanoprost was 18 and 19 mmHg. The slit lamp examination revealed peripheral radial transillumination defects OU (Figure 1).

**Figure 2:**
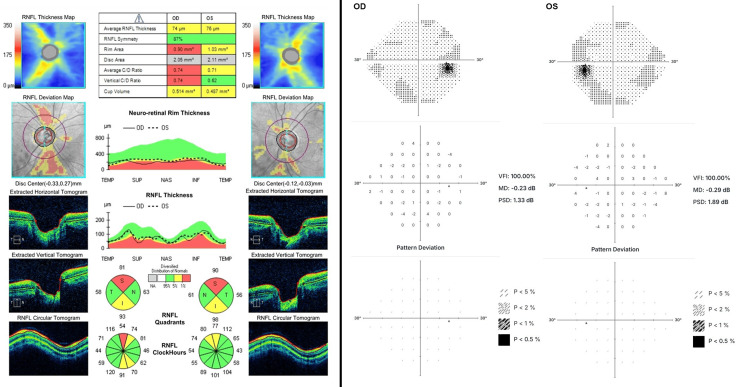
Preoperative macular OCT (left) and 24-2 visual fields (right). Superior and inferior RNFL thinning consistent with glaucoma can be observed OCT: optical coherence tomography; RNFL: retinal nerve fiber layer; OD: oculus dexter; OS: oculus sinister; VFI: visual field index; MD: mean deviation; PSD: pattern standard deviation; INF: inferior; NAS: nasal; SUP: superior; TEMP: temporal

Further testing revealed an average RNFL thickness of 69 OU with superior and inferior thinning in OU on nerve optical coherence tomography (OCT) (Figure 2). Visual field testing was grossly full OU (Figure 2). The combination of peripheral iris defects, IOP in the high teens while on latanoprost, and thinning of the RNFL led us to reaffirm the previous diagnosis of PDG.

His corneal topography and tomography were normal with low ectasia risk and minimum pachymetry of 529 OD and 534 OS, documented via a Pentacam (OCULUS, Arlington, WA). Additionally, he had a white-to-white distance of 12.4 mm OU and an anterior chamber (AC) depth of 3.68 mm OD and 3.64 mm OS measured with a Pentacam. We discussed EVO ICL (STAAR Surgical, Monrovia, CA) as a treatment option for correcting myopia and providing a physical barrier between the zonules and the posterior pigment epithelium of the iris. We also discussed the risk of worsening the pigment dispersion and the potential need for removal. The patient was amenable to trying EVO ICL. Surgery was completed without complication. Ultrasound biomicroscopy (UBM) using Sonomed (Westbury, NY) and ICL Guru was utilized to calculate and predict the vault and angle. A 13.2 mm OD size was selected, with a predicted vault of 537 µm and a 30° angle. A size of 13.2 mm was selected OS, with a predicted vault of 608 µm and a 30° angle.

**Figure 3:**
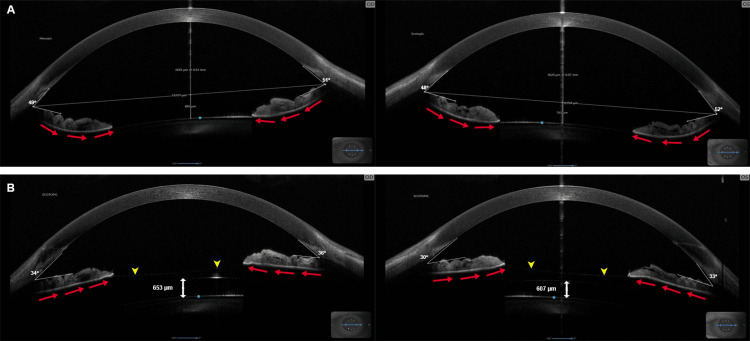
Anterior chamber imaging using MS-39 anterior segment OCT before (A) and one-month after (B) ICL implantation. White lines indicate the trabecular iris angle, labeled in degrees. Red arrows in (A) and (B) indicate the angle of the iris. Blue asterisks indicate the position of the crystalline lens. White arrows in (B) indicate the vault or distance from the bottom of the ICL to the crystalline lens, labeled in micrometers. Yellow arrows indicate the top of the ICL, which is in contact with the posterior iris OCT: optical coherence tomography; ICL: intraocular collamer lens

On post-op day 1, the patient’s vision was 20/20+2 OD and 20/15 OS uncorrected with IOPs of 11 and 9 mmHg by Goldmann tonometry (GT), respectively. His vault (central measurement between the anterior capsule of the crystalline lens and posterior aspect of the ICL) was 744 µm OD and 766 µm OS. He discontinued latanoprost the day of surgery. At post-op month (POM) 1, reconfiguration of the iris contour was observable (Figure 3) with a vault of 653 µm OD and 607 µm OS.

**Figure 4:**
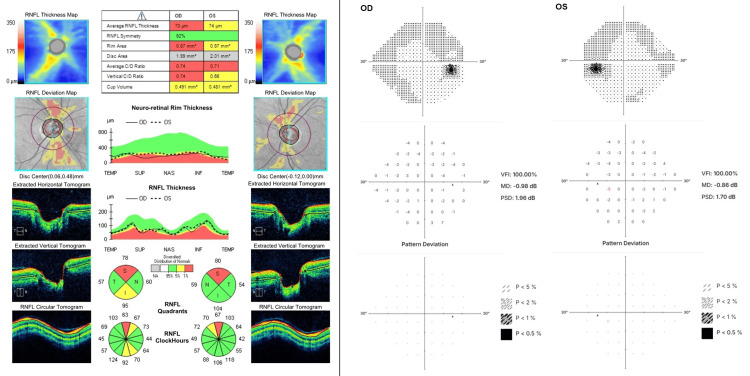
Postoperative month six OCT (left) and 24-2 visual fields (right). The patient has maintained preoperative global RNFL thickness in the postoperative period OCT: optical coherence tomography; RNFL: retinal nerve fiber layer; OD: oculus dexter; OS: oculus sinister; VFI: visual field index; MD: mean deviation; PSD: pattern standard deviation; INF: inferior; NAS: nasal; SUP: superior; TEMP: temporal

**Figure 5:**
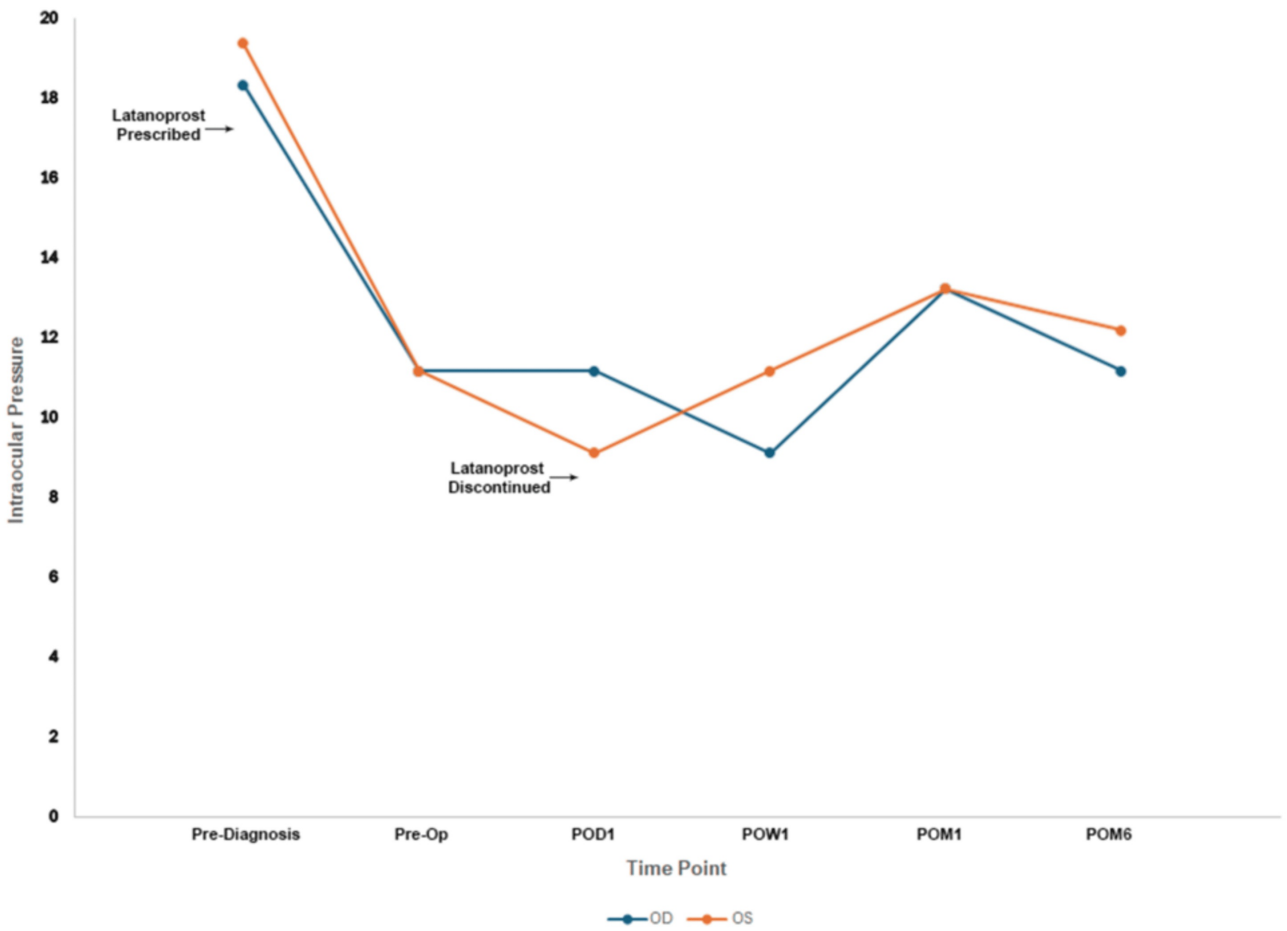
Intraocular pressure readings throughout the patient’s clinical course OD: oculus dexter; OS: oculus sinister; POM: post-op month

Angles OD were 34° in the temporal quadrant and 36° in the nasal quadrant. Angles OS were 33° in the temporal quadrant and 30° in the nasal quadrant. IOP was 13 OU by GT. Visual acuity was 20/15+1 OD and 20/12.5+2 OS uncorrected. At POM 6, visual acuity was 20/12.5+2 OU uncorrected, and IOP was 11 OD and 12 OS by GT, with no changes to visual fields and preserved global RNFL thickness (Figures 4, 5).

## Discussion

Our results suggest ICLs may have therapeutic applications in the setting of preexisting PDS/PDG. We observed a stable reduction in contact between the posterior iris and crystalline lens post-ICL implantation on OCT (Figure 3), suggesting the ICL can act as a barrier to produce separation between these structures. The patient's postoperative course was typical for an EVO ICL patient. While their initial vault was slightly elevated OS, by postoperative month 1, the vault was within the standard range OU. Additionally, within the six-month follow-up period of this study, no acute glaucoma events were reported, and pressures remained within normal OU, suggesting iris-lens contact was a major driver of their symptoms and the EVO ICL may have conferred a therapeutic effect (Figure 5). Our findings agree with a report by Rodriguez-Una et al., where an ICL was implanted in a patient PDS. Similar to our case, ICL insertion led to a change in iris morphology from concave to parallel, reducing the IOP without medication [8]. In their case, preoperative IOP was 27 mmHg OU. One eye was treated with neodymium: yttrium-aluminum-garnet (Nd:YAG) LPI OD and EVO ICL OS. The IOP was reduced to 18 mmHg OU at the six-month postoperative period, and improvement of concave iris configuration was noted OU, suggesting comparable performance between the two treatment options. Additionally, a study on 20 patients with concave irises treated with EVO ICL showed statistically significant decreases in iris curvature postimplantation [9]. These patients showed no statistically significant differences in postoperative iridocorneal angle or posterior chamber angle compared with 20 healthy controls without a concave iris treated with EVO ICL. No patients in this study developed pigment dispersion symptoms preoperatively or postoperatively during the approximately one-year follow-up period. The authors also concluded that the risk of intraocular pigment dissemination due to iris concavity may be reduced by changes in iris configuration [9].

Previous reports in the literature have reported pigment dispersion as a postoperative complication of ICL implantation [10-12]. However, many of these cases were documented with older ICL models, notably the Visian ICL (STAAR Surgical, Monrovia, CA). Unlike the current EVO ICL model, these ICLs were not constructed with central ports to facilitate aqueous flow. Prior to this modification, ICL implantation was often paired with peripheral iridotomy to help prevent complications such as increased pressure and PDS [13]. In several of these reports, the postoperative vault was not directly stated, meaning the contribution of iatrogenic error is unclear. Several trials demonstrate the safety and efficacy of EVO ICL with extremely low levels of postoperative complications, including pigment dispersion [9,14,15].

Accurate prediction of the postoperative vault is essential to ensure the success of ICL surgery. Determining whether a PDS patient may be a good candidate for ICL implantation must consider preoperative AC characteristics, as ICLs can lead to marked changes in AC volume and depth [16]. Furthermore, there is potential for positional shifts in ICL footplates, leading to insertion under the ciliary body [17]. This positioning may subsequently promote IOL-iris contact and worsening pigment dispersion. Such shifts are more likely to occur in eyes with thinner ciliary bodies and wider trabecular-ciliary angles [16]. Several studies have explored the development of predictive models to determine postoperative vault [18-20]. Our group has developed deep learning models to accurately predict postoperative vault and determine ICL sizing using UBM and OCT [21,22]. How these models can be optimized to assist in selecting ICLs in PDS patients may be worth investigating. Variations in vault are well tolerated by patients, and major fluctuations are not expected postoperatively beyond six months [15]. Using UBM combined with artificial intelligence predictive models from ICL Guru, we were able to closely predict our postoperative vault and angles from preoperative measurements. As a result, we were able to accurately size both ICLs to create an appropriate vault and change in iris configuration. Furthermore, the postoperative progression of the vault was consistent with the vault changes of -19.53 ± 111.28 μm/month between post-op one week and one month, as proposed in Lin et al. [23]. The patient is being closely followed with excellent stability in IOP control without any significant progression on glaucoma imaging.

## Conclusions

PDS is a condition where lens zonules cause the release of iris pigment, resulting in elevated IOP due to the occlusion of the trabecular meshwork. The EVO ICL may have a therapeutic role in managing pigment dispersion by changing the iris configuration and creating a mechanical barrier between the zonules and posterior iris. Additionally, the EVO exhibits a high safety profile compared to previous ICL models. In this report, a patient on latanoprost did well after discontinuing the drops, with no worsening of transillumination defects or changes in the optic nerve or visual field at six months after ICL implantation. However, the results of this study are based on a single case and are limited by the relatively short follow-up period for tracking postoperative stability and potential disease progression. To determine the general impact of this intervention, larger studies with longer follow-up periods are needed.
